# The efficacy and safety of high-dose nonsedating antihistamines in chronic spontaneous urticaria: a systematic review and meta-analysis of randomized clinical trials

**DOI:** 10.1186/s40360-023-00665-y

**Published:** 2023-04-06

**Authors:** Xianjun Xiao, Peiwen Xue, Yunzhou Shi, Junpeng Yao, Wei Cao, Leixiao Zhang, Zihao Zou, Siyuan Zhou, Chuan Wang, Mingling Chen, Rongjiang Jin, Ying Li, Qianhua Zheng

**Affiliations:** 1 School of Health Preservation and Rehabilitation, Chengdu University of Traditional Chinese Medicine, Chengdu, 610000 Sichuan China; 2 Acupuncture and Tuina School, Chengdu University of Traditional Chinese Medicine, No. 37 Shierqiao Road, Jinniu District, Chengdu, 610075 Sichuan China; 3 Department of Integrated Traditional and Western Medicine, West China Hospital, Sichuan University, Chengdu, 610000 Sichuan China; 4 Acupuncture department, Wuhan NO.1 Hospital, Wuhan, 430000 Hubei China; 5 Dermatological Department, Affiliated Hospital of Chengdu University of Traditional Chinese Medicine, Chengdu, 610000 Sichuan China

**Keywords:** Nonsedating antihistamines, Chronic spontaneous urticaria, Safety, High-dose, Systematic review and meta-ananlysis

## Abstract

**Background:**

Standard doses of second-generation H_1_-antihistamines (sgAHs) as first-line treatment are not always effective in treating chronic spontaneous urticaria (CSU), and hence an increase in the dose of sgAHs is recommended. However, literature evaluating the efficacy and safety of this treatment remains inconclusive, highlighting the need for a systematic review and meta-analysis. The aim of this systematic review and meta-analysis was to evaluate the efficacy and safety of high-dose sgAHs compared with standard-dose sgAHs in treating CSU.

**Methods:**

A systematic literature search of double-blind, randomized controlled trials (RCT) utilizing multiple doses of sgAHs was performed by searching the electronic databases Medline, Embase, PsycInfo, Cochrane databases, and Web of Science. Bibliographies were also manually searched. The Cochrane Risk of Bias Tool for assessing risk of bias was used to assess the quality of randomized controlled trials (RCTs). Two reviewers screened studies, extracted data, and evaluated the risk of bias independently. The response rate, the number of adverse events, somnolence, and withdrawal due to adverse events were extracted from each article. The data were combined and analyzed to quantify the safety and efficacy of the treatment. RevMan (V5.3) software was used for data synthesis.

**Results:**

A total of 13 studies were identified, seven of which met the eligibility criteria for the meta-analysis. Our pooled meta-analyses showed that high-dose sgAHs was associated with a significantly higher response rate than standard-dose (RR 1.13, 95% CI 1.02 to 1.26; *P* = 0.02). Conversely, high doses of sgAHs were associated with significantly higher somnolence rates than standard dose (RD 0.05, 95% CI 0.01 to 0.09; *P* = 0.02). There was no significant difference in adverse events or withdrawal due to adverse events between standard- and high-dose treatments.

**Conclusions:**

Our analyses showed that a high dose of sgAHs (up to two times the standard dose) might be more effective than a standard dose in CSU treatment. High-dose and standard-dose sgAHs showed similar adverse events, except for somnolence, where incidence was found to be dose-dependent in some studies. However, given the limited number of studies, our meta-analysis results should be interpreted with caution.

**Supplementary Information:**

The online version contains supplementary material available at 10.1186/s40360-023-00665-y.

## Introduction

Chronic spontaneous urticaria (CSU), also known as chronic idiopathic urticaria, is a condition characterized by the occurrence of spontaneous wheals, angioedema, or both for more than six weeks [1]. The prevalence of chronic urticaria around the world is estimated to be in the range of approximately 0.1% to 1.4%, and its prevalence appears to be increasing [2, 3]. CSU patients often experience numerous distressing symptoms, including sleep disturbances, fatigue, and psychological distress, leading to a profound reduction in their quality of life [3–6] and a substantial burden for health care systems [7–9].

CSU is typically managed using second-generation H_1_-antihistamines (sgAHs) [3]. The European [1] and American guidelines [10] recommend the use of sgAHs at licensed doses as the first-line treatment for CSU. In CSU patients with insufficiently controlled symptoms, guidelines [1, 10] recommend increasing the dose of sgAHs as a second-line treatment. Most studies on CSU reported on the safety and efficacy of standard-dose sgAHs, while studies evaluating the impact of high-dose sgAHs are mostly small and with low quality. Therefore, evidence for the high-dose of sgAHs in CSU is still limited. One straightforward approach to overcome the limitations of current studies is to combine available data through a meta-analysis [11]. Guillén-Aguinaga et al. [12] presented a meta-analysis with a focus on sgAHs dosing for CSU. The study found that updosing sgAHs significantly improved control of pruritus but not the number of wheals. However, due to the significant heterogeneity and weakness of the studies, it was difficult to reach a final conclusion [12]. As a result, some experts are still concerned that updosing sgAHs might increase adverse events [13, 14]. Furthermore, the study by Guillén-Aguinaga et al. [12] did not evaluate the safety of using high-dose sgAHs, highlighting the need of a meta-analysis. We therefore performed a meta-analysis of randomized controlled trials (RCTs) to evaluate the efficacy and side effects of high-dose sgAHs in the treatment of CSU, in order to provide new evidence for its clinical application.

## Materials and methods

The protocol for this review study was registered in the International Prospective Register of Systematic Reviews (PROSPERO) as CRD42020195864 and followed the Preferred Reporting Items for Systematic Reviews and Meta-analysis recommendations [15]. A systematic literature search was performed using [16]. The search strategy included all published articles up to Feb 2023 and utilized the keywords "randomized controlled trials", "urticarial", "hives", "h1 antihistamine", and "second generation h1 Antihistamine"(Detailed search strategy was in supplementary materials). Furthermore, the bibliographies of any identified RCT and review articles were also analyzed to identify additional published or unpublished data.

### Eligibility criteria for systematic review

To be eligible for systematic review: (1) All double-blind RCT of patients with CSU that compared two or more fixed-doses sgAHs in their treatment groups (i.e. an active drug with placebo, or two or more doses of an active drug with or without placebo), (2) the study reported any of the following outcomes: the response rates (defined as pruritus symptoms reduction higher than 50%, or at least a moderate to very good global symptom improvement.); MPS (mean pruritus score, reflecting the overall situation of pruritus); MNW (mean number of wheals, reflecting the overall situation of wheals); MTSS (calculated as the sum of MPS and MNW, reflecting the overall situation of urticaria symptoms), DLQI (dermatology life quality index) and/or adverse events of the treatment; moreover, the eligible studies did not need to report all of the aforementioned outcomes but had to report response rates or adverse events, (3) Only articles published in English were included.

Studies were excluded if the full-text was inaccessible or if they had insufficient data for data pooling and analysis. All case reports, case series, observational studies, review articles, in vitro studies, comments, and replies were also excluded.

### Study selection

The search result was evaluated by two independent reviewers (YZS and LXZ), and any disagreements were resolved by discussion with a third reviewer (XJX).

### Data extraction and bias assessments

The following information was independently extracted from the full text by two reviewers (WC and RJ): first author, year of publication, sex of patients, the number of sgAHs-treated patients, dose and treatment duration of sgAHs, outcome measurements, treatment response rates and change in CSU activity after treatment with sgAHs, as well as numbers of adverse events.

The quality and risk of bias of the included RCT studies were assessed using the Cochrane collaboration tool [17]. If the data in the study were incomplete, the original author was first contacted to obtain the corresponding data. Alternatively, the change in the response rate score after treatment from baseline was calculated using the formula recommended by the Cochrane handbook [17]. When the data were only presented in graphs, Adobe Photoshop (Adobe, Inc., San Jose, CA) was utilized to extract data [18, 19]. Studies were excluded if any of the above methods failed to provide sufficient data for analysis.

### Statistical analysis

Data collection and analysis were performed using the RevMan V5.3 statistical software provided by the Cochrane collaboration. Random effects models were used for the meta-analysis because of the wide heterogeneity in the design, populations, and sample size between studies [20]. If the quantitative analysis was not appropriate, a descriptive analysis was provided. For continuous data, mean difference and 95% confidence interval (CI) were used to measure the treatment effect. For dichotomous data, risk ratio (RR) with 95% CI were used to measure the treatment effect. In the case of studies with zero events in both arms, the risk difference (RD) was calculated [21]. The researcher agreement and a meta-analysis manual for the three-arm RCT of the Cochrane alliance were used to compare the outcomes in the two control groups [17].

## Results

**Fig. 1:**
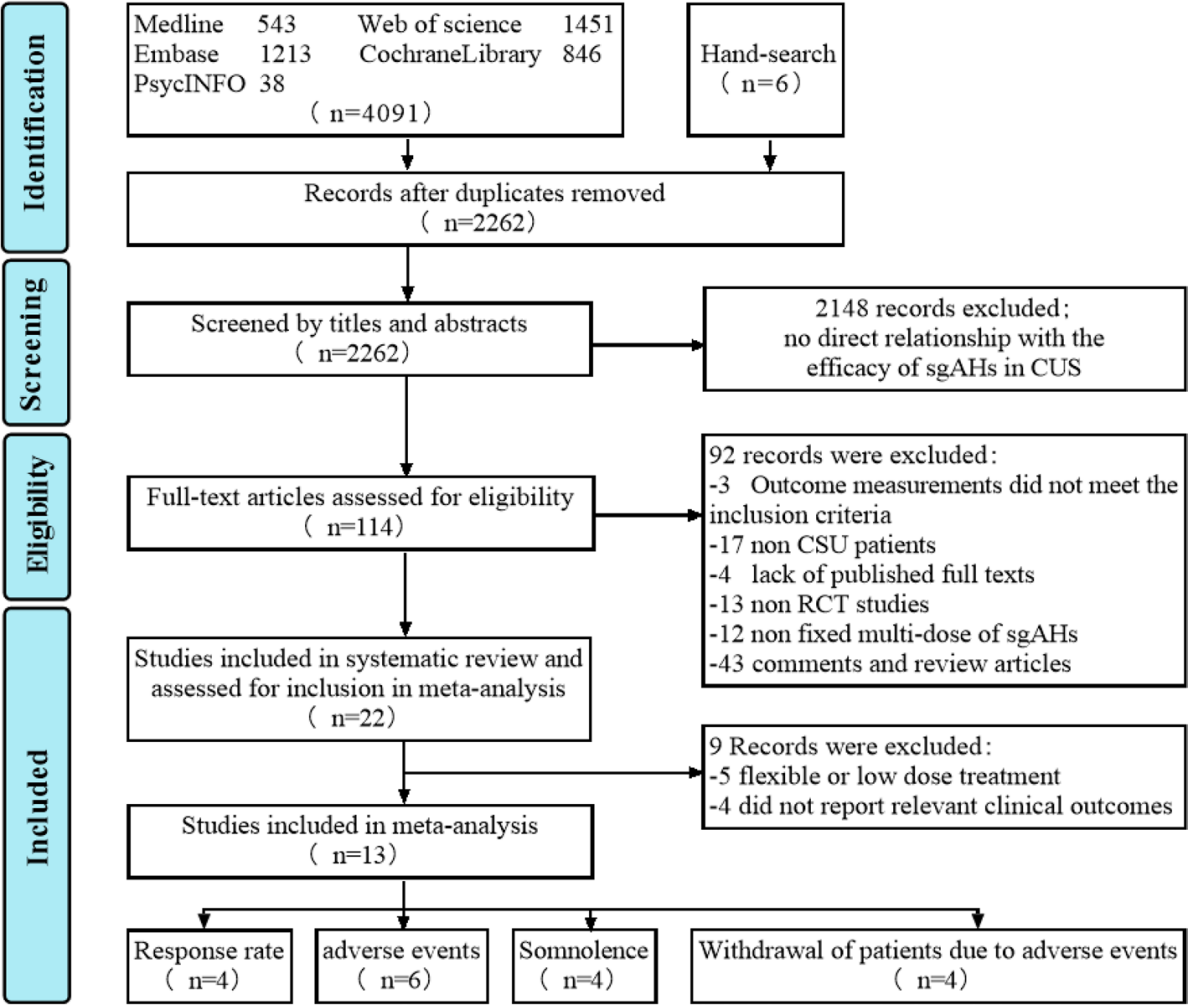
Flow diagram illustrating the search strategy used to identify suitable studies

Through the literature search, a total of 4091 potentially eligible research articles were identified from the following databases (Fig. 1): Medline (*n* = 543), Embase (*n* = 1213), Cochrane library (*n* = 846), PsycInfo (*n* = 38) and Web of Science (*n* = 1451). Six additional articles were identified by manually searching the bibliographies of the articles of interest. Thirty RCTs, one triple-blind, one quadruple-blind and eleven double-blind, were finally included in the meta-analysis [22–34].

**Fig. 2:**
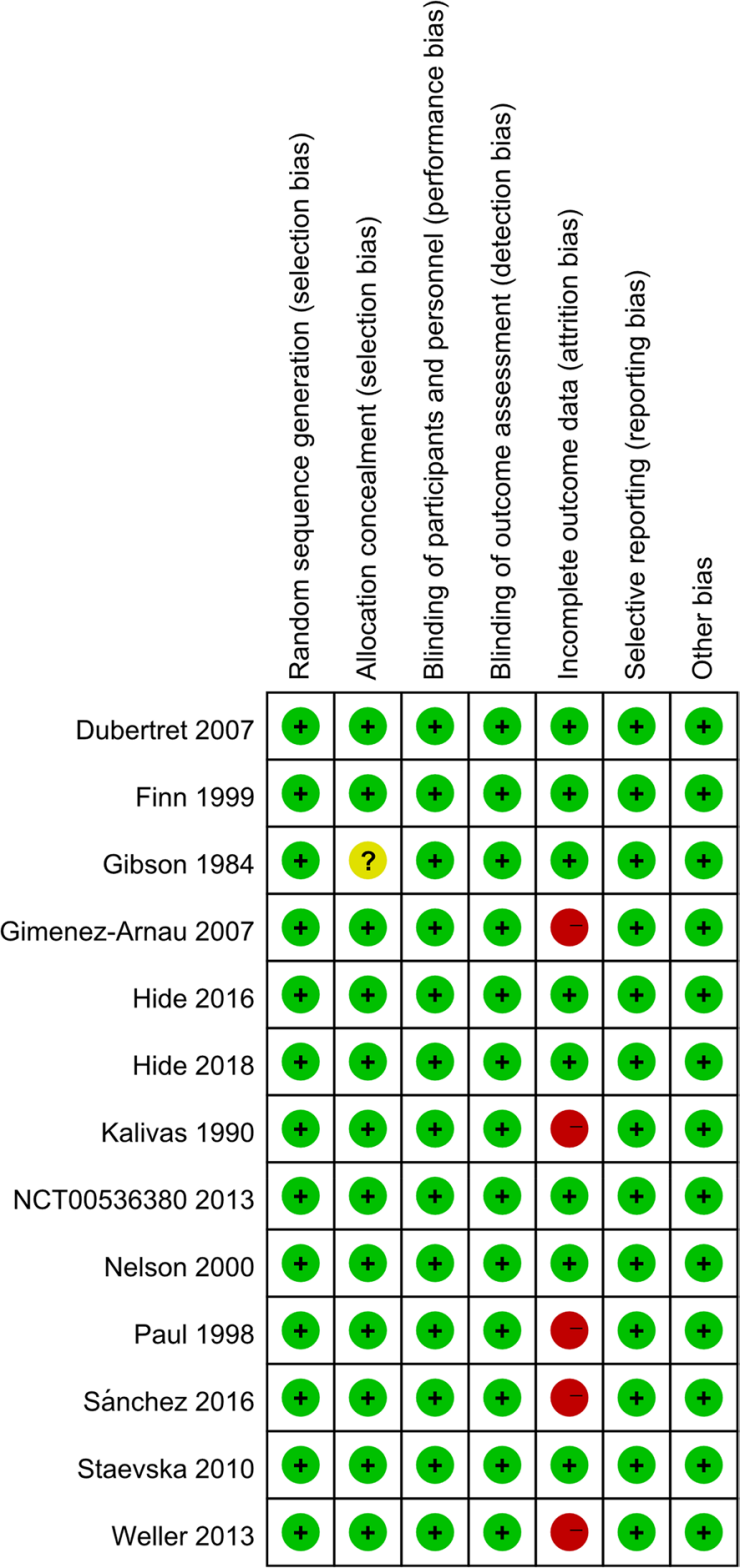
Risk of bias of the included studies

Among the 3079 patients with CSU (Table. 1), sex data were reported by 12 of the studies (1314 male and 1550 female patients) conducted in eight countries (Spain, the United States, Germany, United Kingdom, Japan, Colombia, Bulgaria, and France). Different types of sgAHs including fexofenadine, rupatadine, bilastine, desloratadine, cetirizine, levocetirizine, ebastine, and acrivastine were administered to 880, 586, 229, 413, 99, 40, 30, and 20 patients with CSU, respectively. Fexofenadine was the most studied treatment (4/13 studies) [22, 24, 29, 30]. The age of the patients ranged from 12 to 74 years. The evaluated studies analyzed different treatment outcomes. Four studies evaluated the response rates [22, 23, 28, 33], six studies evaluated adverse events [22–24, 28, 29, 34] and four studies analyzed the somnolence [22, 23, 28, 33] and withdrawal of patients (due to adverse events) of sgAHs treatment [24, 28, 29, 34]. The risk of bias of the included studies is presented in Fig. 2.

**Table 1:** Characteristics of studies included in the systematic review

Study	Sample Size (female,%)	Age	Course of the disease (week)	Outcome measurement	Intervention	TD	Definition of response	Response rate^a^, no. (%)	Adverse events, no. (%)	Somnolence (%)	Main outcome
Gimenez-Arnau et al. (2007) [23]	329 (68.39%)	12–65	≥ 6	5-point rating score ^b^ (MPS, MNW, MTSS); 5-point rating score ^c^ (Global efficacy); DLQI	Rupatadine 10 mg Rupatadine 20 mg Placebo	6 weeks	pruritus symptoms reduction higher than 50%	72 (65.5%) 79 (73.15%) 51 (45.9%)	13 (11.6%) 18 (16.5%) 13 (11.5%)	3 (2.7%) 9 (8.3%) 6 (5.3%)	Rupatadine 10 mg has an overall better AE profile than rupatadine 20 mg. Rupatadine 10 mg is the preferred dose of choice for patients with CIU
Finn et al. (1999) [24]	439 (74.26%)	12–65	≥ 6	5-point rating score ^b^ (MPS, MNW, MTSS), Sleep and daily activities^d^	Fexofenadine HCl 20 mg bid Fexofenadine HCl 60 mg bid Fexofenadine HCl 120 mg bid Fexofenadine HCl 240 mg bid Placebo	4 weeks	NA	NA	67 (71%) 51 (57%) 60 (65%) 50 (59%) 61 (66%)	NA	Fexofenadine 60 mg bid had a larger treatment effect than fexofenadine 20 mg bid. All doses were well tolerated, with safety profiles similar to that of placebo
Gibson et al. (1984) [25]	20 (60%)	19–74	≥ 4	5-point rating score^b^ (pruritus, wheals, Discomfort VAS^e^	Acrivastine 4 mg tid Acrivastine 8 mg tid Placebo tid	5 days	NA	NA	4 (20%) 8 (40%) 6 (30%)	2 (10%) 7 (35%) 3 (15%)	Acrivastine 8 mg was significantly better than Acrivastine 4 mg in improving itching and whealing
Nelson et al. (2000) [29]	418 (70.10%)	12–65	≥ 6	5-point rating score ^b^ (MPS, MNW) sleep and daily activities^d^	fexofenadine HCl 20 mg bid fexofenadine HCl 60 mg bid fexofenadine HCl 120 mg bid fexofenadine HCl 240 mg bid Placebo	4 weeks	NA	NA	NA	NA	Fexofenadine HCl significantly reduced pruritus severity, number of wheals, and twice-daily doses of 60 mg or greater were most effective
Paul et al. (1998) [22]	208 (57.69%)	≥ 18	≥ 6	4-point rating scoref(MPS); 5-point rating score^b^ (MNW); TSS; Sleep and daily activities^d^; Medication effectiveness^i^	Fexofenadine HCI 60 mg Fexofenadine HCI 120 mg Fexofenadine HCI 180 mg Fexofenadine HCI 240 mg Placebo	6 weeks	rated the effectiveness of the medication as good, very good or excellent	25 (63%) 18 (50%) 30 (64%) 21 (55%) 19 (41%)	7 (18%) 9 (26%) 13 (28%) 10 (26%) 15 (33%)	0 (0%) 0 (0%) 0 (0%) 0 (0%) 0 (0%)	There was no significant difference between the 180 mg/day and the 240 mg/day doses. The authors recommend fexofenadine HCI 180 mg/day as the optimal dose for the treatment of CSU
Dubertret et al. (2007) [33]	277 (72.92%)	12–65	≥ 6	5-point rating score^b^ (MPS, MNS, MTSS); sleep and daily activities^d^; Global efficacy^c^	Rupatadine 5 mg Rupatadine 10 mg Rupatadine 20 mg Placebo	4 weeks	0: worse 1: unchanged 2:slight improvement 3:good improvement	37 (54.24%) 42 (57.04%) 49 (72.80%) 17 (25.04%)	NA	3 (4.29%) 4 (5.41%) 14 (21.43%) 2 (2.90%)	Rupatadine 10 mg and 20 mg provides rapid and long-lasting relief from pruritus, in CSU
Weller et al. 2013 [26]	29 (55.17%)	21–65	NA	UAS7; Discomfort VAS^e^; Area size of wheals;	Desloratadine 5 mg (on demand) Desloratadine 20 mg (on demand)	21 days	NA	NA	0 (0%) 0 (0%)	0 (0%) 0 (0%)	The beneficial effects of desloratadine on existing wheals (on-demand treatment) seem to be low
Sánchez et al. (2016) [30]	180 (55.17%)	12–50	≥ 6	UAS, DLQI	First 4 weeks: cetirizine 10 mg Fexofenadine 180 mg bilastine 20 mg Desloratadine 5 mg Ebastine 20 mg Placebo Second 4 weeks: 2 ~ fourfold dose based on the original drug	8 weeks	Controlled: (DLQI ≤ 5) Moderate: (DLQI 6–9) Uncontrolled: (DLQI ≥ 10)	onefold: 88 (58.7%) 2 ~ fourfold: 115 (76.7%)	87 (58%)	onefold: 43 (28.6%) ~ 2–fourfold: 34 (22.6%)	The safety and efficacy of the 5 antihistamines were similar. After updosing, rates of disease control increased from 58.7% to 76.7%
Hide et al. (2016) [27]	294 (73.81%)	18–74	≥ 4	4-point rating score^f^ (wheals); 5-point rating score ^b^ (pruritus); TSS; DLQI; Overall improvement score^g^	Bilastine 10 mg Bilastine 20 mg Placebo	2 weeks	1.markedly improved; 2.moderately improved; 3.mildly improved; 4.no change; 5.exacerbated 6.not evaluable	84 (84.8%) 74 (74.7%) 30 (31.6%)	24 (24.0%) 14 (13.9%) 20 (19.4%)	2 (2.0%) 0 (0.0%) 3 (2.9%)	Bilastine 20 and 10 mg once a day was effective and tolerable in Japanese patients with CSU.
Hide et al. (2019) [28]	276 (65.94%)	12–64	≥ 4	5-point rating score ^b^ (TPS, NWS, RDS); PWS, DLQI; Overall improvement score ^h^	Rupatadine 10 mg Rupatadine 20 mg Placebo	2 weeks	1.extremely improved 2.very improved 3.moderately improved 4.no change 5.worsened	68 (74.8%) 71 (78.1%) 29 (30.8%)	19 (20.9%) 16 (17.4%) 8 (8.5%)	10 (11.0%) 9 (9.8%) 0 (0%)	The optimal rupatadine dose was 10 mg once daily. The dose can be safely increased to 20 mg once daily,
Staevska et al.2010 [31]	80 (62.67%)	19–67	≥ 6	CU-Q2oL; Discomfort VAS^e^; ASST	Levocetirizine (5 mg/1^st^ wk, 10 mg/2^nd^ wk, 20 mg/3^rd^ wk) Desloratadine (5 mg/1^st^ wk, 10 mg/2^nd^ wk, 20 mg/3^rd^ wk) levocetirizine 20 mg switch desloratadine 20 mg (4^th^ wk)	4 weeks	Patients who had no urticarial lesions and no pruritus for the last 3 days of treatment were considered to be symptom-free	Levocetirizine/ Desloratadine: 1^st^ wk 9/4 2^nd^ wk 8/7 3^rd^ wk 5/1 4^th^ wk 7/0	6 (15%) 11 (27.5%)	No detailed data	Levocetirizine and desloratadine, to up to 4 times the conventionally prescribed doses increases the control of urticaria symptoms in approximately 75% of patients without compromising somnolence or safety. Levocetirizine was more effective drug in the course of treatment with 5-mg to 20-mg daily doses
Kalivas et al. 1990 [32]	215 (NA)	≥ 12	≥ 6	Four-point rating score^f^ (wheals, pruritus and number of episodes); Global efficacy^c^	Cetirizine 5 mg ~ 20 mg; Hydroxyzine 25 ~ 75 mg; Placebo	4 weeks	NA	NA	No detailed data	15 (21.7%) 26 (36. 1%) 10 (13.5%)	cetirizine has a greater safety margin over the older parent drug hydroxyzine
NCT00536380 2013 [34]	314(66.56%)	≥ 18	≥ 6 weeks	UAS	desloratadine 5 mg desloratadine 10 mg desloratadine 20 mg	4 weeks	NA	NA	7(6.6%) 5(4.8%) 2(1.9%)	NA	There was no significant difference in UAS scores improvement between the 5 mg, 10 mg and 20 mg desloratadine groups

*Abbreviations:* *TD* Treatment duration, *DLQI* Dermatology life quality index, *VAS* Visual analogue scale, *MPS* Mean pruritus score, *MNW* Mean number of wheals, *MTSS* Calculated as the sum of MPS and MNW, *NA* data were not available in the study, *TSS* The sum of the wheal and pruritus scores, *TPS* Total pruritus score, *NWS* Number of wheals score, *RDS* Rash duration score, *PWS* The sum of pruritus and number of wheals score, *UAS* Urticaria activity score, *ASST* Autologous serum skin test, *CU-Q2oL* Chronic urticaria quality of life questionnaire

^a^Response, pruritus symptoms reduction higher than 50%, or overall improvement rated at least moderately/very improved

^b^ Five-point rating score (0–4): 0 = no symptom to 4 = the worst symptom, the higher the score, the worse the symptoms

^c^ Global efficacy (0–4): 0 = worse to 4 = excellent improvement, the higher the score, the better the symptoms

^d^ sleep and daily activities: (0–3): 0 = none, 1 = mild, 2 = moderate, and 3 = severe

^e^Discomfort VAS: patients evaluated their drowsiness, itching and severity of symptoms by marking along a 0–100 mm long horizontal line (0 = min to 106 = max)

^f^Four-point rating score (0–3): 0 = no symptom to 3 = the worst symptom, the higher the score, the worse the symptoms;

^g^ Five-point rating score (1–5): 1 = markedly improved to 5 = exacerbated, the higher the score, the worse the symptoms;

^h^ Overall improvement score (1–6): 1 = extremely improved, 2 = very improved, 3 = moderately improved, 4 = no change, 5 = worsened, and 6 = not evaluable

^i^ medication effectiveness (0–4, 0 = excellent to 4 = none)

### High-dose versus standard-dose sgAHs

#### Response rate

**Fig. 3:**
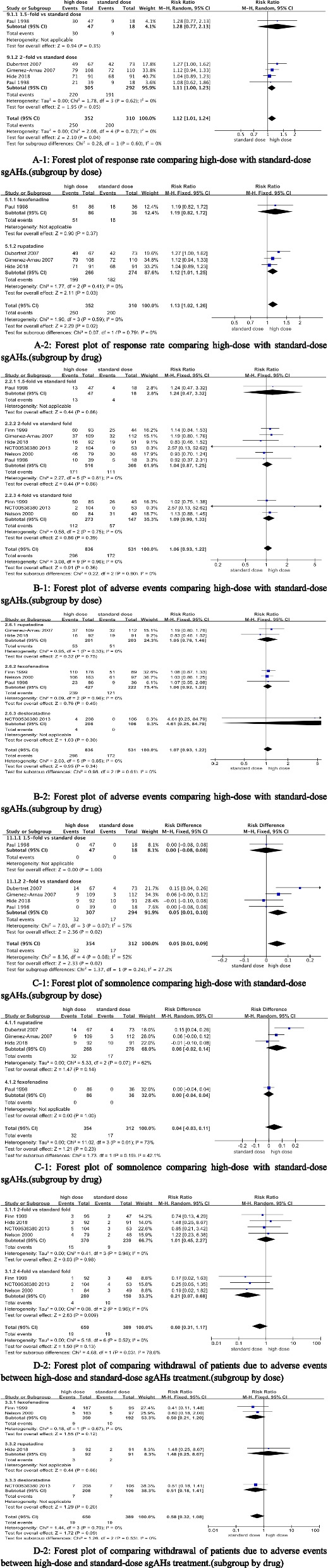
**A-1** Forest plot of response rate comparing high-dose with standard-dose sgAHs.(subgroup by dose). **A-2** Forest plot of response rate comparing high-dose with standard-dose sgAHs.(subgroup by drug). **B-1** Forest plot of adverse events comparing high-dose with standard-dose sgAHs.(subgroup by dose). **B-2** Forest plot of adverse events comparing high-dose with standard-dose sgAHs.(subgroup by drug). **C-1** Forest plot of somnolence comparing high-dose with standard-dose sgAHs.(subgroup by dose). **C-2** Forest plot of somnolence comparing high-dose with standard-dose sgAHs.(subgroup by drug). **D-1** Forest plot of comparing withdrawal of patients due to adverse events between high-dose and standard-dose sgAHs treatment.(subgroup by dose). **D-2** Forest plot of comparing withdrawal of patients due to adverse events between high-dose and standard-dose sgAHs treatment.(subgroup by drug)

The response was reported in four studies (Fig. 3A) [22, 23, 28, 33] with a total of 352 patients treated with high-dose and 310 patients treated with standard-dose sgAHs. High-dose sgAHs was associated with a significantly higher response rate when compared with standard dose (RR 1.13, 95% CI 1.02 to 1.26; *P* = 0.02).

#### Adverse events

Adverse events (Fig. 3B) were reported in six studies [22–24, 28, 29, 34] with a total of 1367 patients evaluated (high dose *n* = 836; standard dose *n* = 531). There was no significant difference in patients with CSU who experienced at least one adverse event between high-dose and standard-dose treatments (RR 1.06, 95% CI 0.93 to 1.22; *P* = 0.36).

#### Somnolence

Somnolence (Fig. 3C) was reported in four studies [22, 23, 28, 33] including 666 patients (high dose *n* = 354; standard dose *n* = 312). A high-dose sgAHs was associated with significantly higher somnolence rates when compared with the standard dose (RD 0.05, 95% CI 0.01 to 0.09; *P* = 0.02). Due to the noted significant heterogeneity between the included studies (I^2^ = 52%), the leave-one-out analysis was performed. When the study by Dubertret et al. [33]. was excluded from the analysis, the heterogeneity was significantly reduced (I^2^ = 0%), which suggested that it is a heterogeneous source. When other studies pooled, there was no significant difference in somnolence between high dose and standard dose (RD 0.02, 95% CI -0.02 to 0.06; *P* = 0.36). This indicated that the results were not robust enough and should be treated with caution.

#### Withdrawal of patients due to adverse events

Withdrawal of patients due to adverse events (Fig. 3D) was reported in four studies [24, 28, 29, 34] including 1039 patients (high dose *n* = 650; standard dose *n* = 389). There was no significant difference in the number of patients withdrawing from treatment due to adverse events between high-dose and standard-dose treatments (RR 0.60, 95% CI 0.31 to 1.17; *P* = 0.13).

### Assessment of treatment efficacy

#### Rupatadine

Gimenez-Arnau et al. [23] reported that rupatadine provided over six weeks using doses of 10 mg and 20 mg led to a decrease in the mean pruritus score (MPS) from baseline by 59.5% and 66.1%, respectively. Both doses resulted in a significantly improved MPS score but did not result in a significant reduction in the mean number of wheals (MNW) score when compared with the placebo. Furthermore, both doses of rupatadine effectively relieved the symptoms of CSU after the first administration. The MPS, MNW, and total symptom scores did not differ between doses at different time points. However, 10 mg rupatadine had an overall better adverse event profile when compared with 20 mg rupatadine.

Hide et al. [28] reported a mean total pruritus score (TPS) difference of -1.956 and -2.121 for 10 mg and 20 mg rupatadine, compared with the placebo, respectively. There was no statistically significant difference between 10 and 20 mg rupatadine for TPS and MNW.

Dubertret et al. [33] reported on patients with CSU treated with 5 mg, 10 mg, and 20 mg rupatadine once daily for four weeks. Over the four weeks treatment period, 10 and 20 mg rupatadine resulted in a significant reduction in pruritus severity of 62.7% and 72.3%, respectively, compared with 45.8% with placebo. Rupatadine at 5 mg resulted in a reduction in the pruritus severity of 51.6%. However, this reduction was not statistically significant when compared with the placebo. Rupatadine at 5 mg, 10 mg, and 20 mg led to a decrease in the MNW from baseline by 34.3%, 45.2%, and 57.8%, respectively over the four weeks treatment period, but this reduction was not statistically significant when compared with the placebo, which resulted in a reduction of 30.1%.

#### Fexofenadine

Finn et al. [24] reported that bidaily (bid) doses of fexofenadine at 20 mg, 60 mg, 120 mg, and 240 mg, provided over four weeks, led to a significant reduction in pruritus severity and the number of wheals in CSU patients when compared with the placebo. Efficacy results in reducing pruritus were similar in the 60, 120, and 240 mg groups and better when compared with the 20 mg group. However, the 120 and 140 mg groups resulted in a reduction in MNW and MTSS scores when compared with the 60 mg group, although the difference was not statistically significant.

Nelson et al. [29] reported reductions in pruritus severity from baseline of 19%, 38%, 54%, 43%, and 57% in the placebo, 20, 60, 120, and 240 mg bid fexofenadine dose groups, respectively, as well as reductions in the MNW from baseline of 18%, 35%, 50%, 64%, and 54% in the placebo 20, 60, 120, and 240 mg bid fexofenadine dose groups, respectively. All fexofenadine HCl doses were statistically superior to placebo in reducing MPS and MNW scores but resulted in increased levels of interference with sleep and daily activities in a significant linear trend with dose.

Paul et al. [22] reported that approximately 73% to 81% of CSU patients receiving 60 mg, 120 mg, 180 mg, and 240 mg of fexofenadine were considered to have a significant improvement in TSS compared with 54% in the placebo group, with the 120 mg and 240 mg doses producing the most significant improvement. When individual fexofenadine groups were compared with placebo, only the 180 mg fexofenadine showed significant reductions in the MNW with a decrease of 0.52 ± 0.19.

#### Acrivastine

Gibson et al. [25] reported that acrivastine at 4 mg and 8 mg significantly reduced symptoms of urticaria compared with placebo. Acrivastine has a rapid therapeutic effect, which reached its peak within two hours.

#### Desloratadine

Weller et al. [26] reported that 5 mg and 20 mg on-demand treatment of desloratadine led to an effective reduction in the hyperthermic skin area, but there was no improvement in wheal area, pruritus, and global efficacy compared with no treatment.

NCT00536380 [34] reported that 5 mg, 10 mg and 20 mg treatment of desloratadine reduced the score of UAS. However, due to poor enrollment (even after extending the enrollment period), only 314 participants (not 600 participants) were randomized to the study and hence the study was inconclusive due to the lacking of statistical power and robustness.

#### Bilastine

Hide et al. [27] reported that Bilastine at 10 and 20 mg administered over a two-week period resulted in a decrease in TSS from baseline of 3.3 and 3.01 respectively, which were significantly better than placebo (with a 1.49 reduction). Bilastine at 10 mg and 20 mg significantly improved wheal and pruritus when compared with placebo. The effectiveness of bilastine once daily could last throughout the day.

#### Cetirizine

Kalivas et al. [32] reported on 69 CSU patients treated with cetirizine once daily for four weeks at a dose of approximately 5 to 20 mg. Cetirizine was better than the placebo at reducing the number and size of lesions, the number of urticarial attacks, and the severity of pruritus.

#### Combined use of* sgAHs*

Staevska et al. [31] studied two groups of CSU patients receiving 5 mg of either desloratadine or levocetirizine in the first week. If this dose was not successful within the next week, the dose was doubled during the following week up to a maximum of four times of the standard dose. The two groups switched the two types of treatment up to a maximum of four times the standard dose of sgAHs in the fourth week. There were significant differences in the number of successful treatments comparing high and standard doses for both levocetirizine and desloratadine. The overall success rate of 22 patients with levocetirizine was significantly higher than the rate of the 12 patients treated with desloratadine at the end of week 3. At the end of the third week, patients who were still symptomatic switched to the opposite drug. Seven patients who did not respond to 20 mg of desloratadine had no more symptoms after taking 20 mg of levocetirizine, while there was no benefit in switching to loratadine in 18 patients who had not been cured with 20 mg of levocetirizine.

Sánchez et al. [30] reported on 150 CSU patients (30 per group) receiving a daily oral standard dose of ebastine (20 mg), bilastine (20 mg), fexofenadine (180 mg), cetirizine (10 mg) or desloratadine (5 mg) over four weeks, respectively. After four weeks, the sgAHs dose was modified up to approximately two or four times the standard dose according to its clinical effectiveness and adverse reactions. There was no significant difference in disease control among the groups. After four weeks of antihistamine treatment using standard doses, the symptoms were completely controlled in 58.7% of patients (*n* = 88) and partially controlled in 30.7% (*n* = 46) of patients. Clinical response in patients with DLQI greater than 5 improved in most patients when the antihistamine dose was increased, with 76.7% (*n* = 115) of patients having their symptoms fully controlled, 15.3% partially controlled (*n* = 23), and 6.7% uncontrolled (*n* = 10).

### Adverse events

A total of seven studies reported adverse events [22–24, 28, 29, 33, 34] while no deaths occurred. Four serious adverse events [23, 24, 33] were reported, but they were not significantly associated with sgAHs treatment. There was no significant difference in the incidence of adverse events between high dose and standard dose (RR 1.06, 95% CI 0.93 to 1.22; *P* = 0. 36) in sgAHs treatment. Somnolence was the most concerning adverse event experienced in high-dose groups, which was reported by four studies [22, 23, 28, 33]. Higher doses of sgAHs were associated with a higher incidence of somnolence when compared with standard dose (RD 0.05, 95% CI 0.01 to 0.09; *P* = 0.02). Headache (16.2%, *n* = 84) was the most common adverse event experience in high-dose groups, followed by upper respiratory infection (10.9%, *n* = 37), somnolence (9.0%, *n* = 32), nasopharyngitis (7.1%, *n* = 32) and gastrointestinal symptoms (8.2%, *n* = 28). Six studies [22, 24, 28, 29, 33, 34] reported on the need to withdraw treatment due to adverse events. Compared with the standard doses, a high-dose treatment did not increase the need to withdraw treatment due to adverse events (*n* = 1039, RR 0.60, 95% CI 0.31 to 1.17; *P* = 0.13) except for fexofenadine. Fexofenadine was the drug with the most reported adverse events in the high-dose group, but rarely including somnolence.

## Discussion

Treatment with sgAHs is the preferred management of CSU as it is safe, convenient, and cost-effective. Both European [1] and American guidelines [10] recommend increasing the dose of sgAHs as a second-line treatment for CSU. However, studies on the efficacy and safety of using a high dose of sgAHs for the treatment of CSU are limited and still inconclusive [35, 36]. European [1], British [37], American guidelines [10], Chinese [38] and Japanese [39] guidelines recommend increasing the dose of sgAHs up two to four times the recommended dose. Higher doses of sgAHs might provide more efficacy, but current data are limited and conflicting for certain agents [10]. Both the European and American guidelines recommend using the lowest number and safest medications to manage CSU [40]. The premise of increasing sgAHs dose is that high dose of sgAHs is more effective than the standard-dose sgAHs. If high-dose sgAHs cannot improve the efficacy, increasing the dose of sgAHs is of little significance, and alternative treatment options should be considered as soon as possible. On the other hand, if the high dose proves to be beneficial, it will provide strong evidence for the development of new consensus guidelines.

We, therefore, performed a systematic review and meta-analysis on the treatment of CSU with high-dose sgAHs to clarify that the efficacy and safety of high-dose sgAHs with a special focus on safety, since a previous meta-analysis conducted by Guillén-Aguinaga et al. [12] only reported on efficacy. The findings of our meta-analysis suggest that high-dose treatment up to a maximum of double the standard dose of sgAHs might provide a better response rate when compared with conventional treatment in patients suffering from CSU. A systematic review of observational studies and RCTs by Iriarte et al. [41] suggested that higher doses of sgAHs for better efficacy in CSU, which is consistent with our conclusions. We differ from them in that Iriarte et al. [41] reviewed and analyzed the safety and efficacy of sgAHs in CSU, whereas we only included RCTs and performed quantitative analysis through meta-analysis. Zhou et al. [42] conducted a meta-analysis on the efficacy and safety of sgAHs in the treatment of CSU, and they found no significant difference in response rates between high and standard doses of sgAHs. Zhou et al. [42] searched the three databases of Pubmed, Embase and Cochrane up to January 2021, and finally included 9 publications. However, we included a total of 13 articles by refining the search strategy and additionally searching Web of science and PsycInfo databases up to February 2023. In addition, the main reason for our different conclusions is that they included a literature [43] comparing levocetirizine 10 mg with a combination of levocetirizine 5 mg and montelukast 10 mg. Notably, our results support current guidelines [1, 10] for the treatment of sgAHs with CSU, where increasing the dose can improve efficacy. High-dose and standard-dose sgAHs showed similar safety profiles. However, this improvement came at the cost of increasing specific adverse events, with somnolence being reported as most distressing for the patient. The overall result of our meta-analysis identified the prevalence of somnolence as being dose-dependent. However, this result seemed to be heavily influenced by one of the Dubertret L's study [33], whereby its exclusion ultimately resulted in no difference in the somnolence incidence between high dose and standard dose of sgAHs. Thus, the finding should be taken with caution.

In our meta-analysis, the response rate using the standard dose was 64.5% and 71.2% in the high-dose treatment. Our results are inconsistent with Guillén-Aguinaga's [12], which may be due to different defining criteria for response rates. Guillén-Aguinaga defined the failed treatment response as an overall symptom improvement of less than 50% or treatment termination due to failure, while we defined the respondent patients according to the overall degree of improvement as indicated in the original text. We are concerned about whether high-dose sgAHs can improve the efficacy of CSU.

There are a number of factors that may lead to poor response following high-dose treatment. CSU is a self-limiting disease, and urticarial activity tends to relapse over time [12, 44]. This implies that the therapeutic effect may be dose- and time-dependent [26, 33], and therefore, continuous and regular medication might provide more effective symptom relief in CSU patients. Furthermore, the findings of the three studies included in our meta-analysis revealed no significant difference between low and standard sgAHs doses (*n* = 415, RR 1.09, 95% CI 0.96 to 1.25; *P* = 0.18). The effect of sgAHs on response rate may not necessarily be linked with the dose, and therefore, further high-quality studies evaluating the impact of dose and time response are needed.

Although treatment with sgAHs may be accompanied by headache, somnolence, nasopharyngitis, and other side effects, these adverse events are reported to be rare, mild, and transient in both high- and standard-dose groups. Somnolence is an adverse event of major concern for both patients and doctors, eventually limiting dose escalation of sgAHs [45]. A total of four studies evaluated the incidence of somnolence, with a total of 63 cases of somnolence (6.3%) being reported after taking sgAHs. The incidence rate was higher in the high-dose group (9%) when compared with standard treatment (5%).

The results of the Cochrane collaboration tool in our study showed that all trials were rated as low risk of bias on selection bias, performance bias, detection bias and reporting bias, except that a study were unclear in allocation concealment [25]. For attrition bias, some of included studies were rated as high risk of bias on items involving incomplete outcome data [22, 23, 26, 30, 32]. Thus, researchers should pay attention to these issues to reduce the risk of bias of randomized controlled trial. In brief, the risk of bias of included studies was low-moderate, indicating that there was certain power to ensure the therapeutic effect.

### Implications for practice

To form the implications for practice and provide strong evidence for the development of new consensus guidelines, we combined the efficacy and safety in randomized clinical trials with into a single overall summary. Collectively, we performed the safety and adverse events evaluation of using high-dose sgAHs for the treatment of CSU. Our study provides comparative data on licensed high-dose sgAHs for guiding treatment selection. In patients with CSU, high doses of sgAHs at up to twice the standard dose may provide better response rates compared to conventional therapy, which enables informed decision making in conjunction with the established treatment guidelines. However, It is not currently possible to confidently rank the efficacy and safety of different high-dose sgAHs due to limited data. Furthermore, particularly, we focused on somnolence, the most distressing of the adverse events of high-dose sgAHs and the results showed that its incidence was dose-dependent. However, due to heterogeneity, the incidence-dose-related results need to be treated with caution. Therefore, the probability of adverse events at higher doses, which may reduce the quality of life of patients, should be considered in decision-making.

### Limitations

Our meta-analysis has some limitations that have to be acknowledged, and therefore, our results should be interpreted with caution. First of all, we did not evaluate all sgAHs. Since different sgAHs are known to exhibit different pharmacokinetic and pharmacodynamic properties, it cannot be ruled out that the use of other antihistamines may lead to different results. The number of studies evaluated in this meta-analysis was small, limiting the generalizability of the research findings. Studies comparing efficacy and safety between standard- and high-dose sgAHs only escalated the dose up to twice the standard dose but not up to four times as recommended by the European guidelines [1]. Moreover, there was considerable variability in the indices used to measure treatment outcomes, and not all outcomes were reported in each study. Therefore, we did not have sufficient data to evaluate the improvement of pruritus, wheals, and DLQI. Furthermore, our study don’t take the details regarding the disease status of CSU patients in inclusion studies into consideration, which will create an situation that the course of the disease itself might be a interference factor of High-dose sgAHs’ efficacy. Consequently, the improvement of CSU may be the result of a combination of time and dose.

## Conclusion

The findings of the meta-analyses showed that high-dose sgAHs (up to two times the standard dose) might be more effective than standard doses in the treatment of CSU. High-dose and standard-dose sgAHs showed similar safety profiles, with the exception of somnolence that might be dose-dependent. However, due to the limited number of studies in our meta-analysis, results should be interpreted with caution.

## Supplementary Information

**Additional file 1.** PRISMA Checklist.

**Additional file 2.** Search strategy.

## Data Availability

All data generated or analysed during this study are included in this published article and its supplementary information files.
