# Supplementary material for: The efficacy and safety of high-dose nonsedating antihistamines in chronic spontaneous urticaria: a systematic review and meta-analysis of randomized clinical trials
Source: BMC Pharmacol Toxicol. 2023 Apr 6;24:23. doi: 10.1186/s40360-023-00665-y (PMC10080829; doi:10.1186/s40360-023-00665-y)
Supplement: Supplementary file 2 — Additional file 2. Search strategy. [file 40360_2023_665_MOESM2_ESM.docx]

**Ovid MEDLINE(R) ALL <1946 to February 16, 2023> （2023-2-17）**

1 exp clinical trial/ 962893

2 exp randomized controlled trials/ 164330

3 exp double-blind method/ 174331

4 exp single-blind method/ 32501

5 exp cross-over studies/ 54694

6 randomized controlled trial.pt. 586804

7 clinical trial.pt. 537120

8 controlled clinical trial.pt. 95190

9 (clinic$ adj2 trial).mp. 802072

10 (random$ adj5 control$ adj5 trial$).mp. 868754

11 (crossover or cross-over).mp. 109083

12 ((singl$ or double$ or trebl$ or tripl$) adj (blind$ or mask$)).mp. 265226

13 randomi$.mp. 1060926

14 (random$ adj5 (assign$ or allocat$ or assort$ or reciev$)).mp. 279385

15 or/1-14 1662932

16 (animals not (human and animals)).sh. 7235797

17 15 not 16 1494487

18 exp *urticaria/ 14330

19 urticaria.ti,ab. 15693

20 hives.ti,ab. 1432

21 or/18-20 23237

22 exp Histamine H1 Antagonists/ 37000

23 h1 antihistamine$.ti,ab. 1015

24 h1 receptor antagonist$.ti,ab. 1840

25 h1 antagonist$.ti,ab. 1152

26 second generation h1 Antihistamine$.ti,ab. 137

27 histamine H1 Antagonists, Non-Sedating.ti,ab. 0

28 Bilastine.ti,ab. or exp Bilastine/ 131

29 Ebastine.ti,ab. or exp Ebastine/ 320

30 Fexofenadine.ti,ab. or exp Fexofenadine/ 966

31 Loratadine.ti,ab. or exp Loratadine/ 1741

32 Desloratadine.ti,ab. or exp Desloratadine/ 614

33 Rupatadine.ti,ab. or exp Rupatadine/ 168

34 Levocetirizine.ti,ab. or exp Levocetirizine/ 451

35 Cetirizine.ti,ab. or exp Cetirizine/ 2068

36 Olopatadine.ti,ab. or exp Olopatadine/ 403

37 Mizolastine.ti,ab. or exp Mizolastine/ 118

38 Epinastine.ti,ab. or exp Epinastine/ 207

39 Emedastine.ti,ab. or exp Emedastine/ 78

40 bepotastine.ti,ab. or exp bepotastine/ 73

41 Acrivastine.ti,ab. or exp Acrivastine/ 100

42 Azelastine.ti,ab. or exp Azelastine/ 731

43 Astemizole.ti,ab. or exp Astemizole/ 860

44 terfenadine.ti,ab. or exp terfenadine/ 2236

45 Clemastine.ti,ab. or exp Clemastine/ 513

46 Setastine.ti,ab. or exp Setastine/ 5

47 or/22-46 40513

48 17 and 21 and 47 543

**Embase <1974 to 2023 February 16> （2023-2-17）**

1 random$.mp. 2162620

2 factorial$.mp. 72740

3 (crossover$ or cross-over$).mp. 136443

4 (placebo$ or PLACEBO).mp. 513526

5 (doubl$ adj blind$).mp. 294153

6 (singl$ adj blind$).mp. 62288

7 (assign$ or allocat$).mp. 678056

8 (volunteer$ or VOLUNTEER).mp. 299417

9 crossover procedure/ 73401

10 double blind procedure/ 205336

11 Randomized Controlled Trial/ 762148

12 Single Blind Procedure/ 50015

13 or/1-12 3119575

14 (animals not (human and animals)).sh. 512

15 13 not 14 3119545

16 exp *urticaria/ 16218

17 urticaria.ti,ab. 25430

18 hives.ti,ab. 2734

19 or/16-18 31499

20 exp Histamine H1 Antagonists/ 221269

21 h1 antihistamine$.ti,ab. 1782

22 h1 receptor antagonist$.ti,ab. 2570

23 h1 antagonist$.ti,ab. 1528

24 second generation h1 Antihistamine$.ti,ab. 224

25 histamine H1 Antagonists, Non-Sedating.ti,ab. 0

26 Bilastine.ti,ab. or exp Bilastine/ 466

27 Ebastine.ti,ab. or exp Ebastine/ 1398

28 Fexofenadine.ti,ab. or exp Fexofenadine/ 5041

29 Loratadine.ti,ab. or exp Loratadine/ 6957

30 Desloratadine.ti,ab. or exp Desloratadine/ 2755

31 Rupatadine.ti,ab. or exp Rupatadine/ 603

32 Levocetirizine.ti,ab. or exp Levocetirizine/ 2225

33 Cetirizine.ti,ab. or exp Cetirizine/ 9055

34 Olopatadine.ti,ab. or exp Olopatadine/ 1394

35 Mizolastine.ti,ab. or exp Mizolastine/ 715

36 Epinastine.ti,ab. or exp Epinastine/ 691

37 Emedastine.ti,ab. or exp Emedastine/ 414

38 bepotastine.ti,ab. or exp bepotastine/ 262

39 Acrivastine.ti,ab. or exp Acrivastine/ 523

40 Azelastine.ti,ab. or exp Azelastine/ 2691

41 Astemizole.ti,ab. or exp Astemizole/ 4128

42 terfenadine.ti,ab. or exp terfenadine/ 6441

43 Clemastine.ti,ab. or exp Clemastine/ 2484

44 Setastine.ti,ab. or exp Setastine/ 19

45 or/20-44 222731

46 15 and 19 and 45 1213

**Web of science (2023-2-17)**

1: clinical trial* (主题) 1542767

2: TS=(research design) 1385386

3: TS=(comparative stud*) 4385992

4: TS=(evaluation stud*) 2438794

5: TS=(controlled trial*) 1207731

6: TS=(follow-up stud*) 1685701

7: TS=(prospective stud*) 1063872

8: TS=(random*) 3450681

9: TS=(placebo*) 362975

10: TS=(single blind*) 124539

11: TS=(double blind*) 428129

12: #11 OR #10 OR #9 OR #8 OR #7 OR #6 OR #5 OR #4 OR #3 OR #2 OR #1 13505292

13: TS=(*urticaria) 40928

14: TS=(urticaria) 40955

15: TS=(hives) 17070

16: #13 OR #14 OR #15 57063

17: TS=(Histamine H1 Antagonists) 25285

18: TS=(h1 antihistamine*) 12407

19: TS=(h1 receptor antagonist*) 15435

20: TS=(h1 antagonist*) 30206

21: TS=(second generation h1 Antihistamine*) 798

22: TS=(Bilastine) 343

23: TS=(Ebastine) 717

24: TS=(Fexofenadine) 2361

25: TS=(Loratadine) 3507

26: TS=(Desloratadine) 1687

27: TS=(Rupatadine) 440

28: TS=(Levocetirizine) 1141

29: TS=(Cetirizine) 4116

30: TS=(Olopatadine) 921

31: TS=(Mizolastine) 299

32: TS=(Epinastine) 493

33: TS=(Emedastine) 199

34: TS=(bepotastine) 237

35: TS=(Acrivastine) 225

36: TS=(Azelastine) 1508

37: TS=(Astemizole) 1562

38: TS=(terfenadine) 3828

39: TS=(Clemastine) 906

40: TS=(Setastine) 50

41: #40 OR #39 OR #38 OR #37 OR #36 OR #35 OR #34 OR #33 OR #32 OR #31 OR #30 OR #29 OR #28 OR #27 OR #26 OR #25 OR #24 OR #23 OR #22 OR #21 OR #20 OR #18 OR #19 OR #17 42264

42: #41 AND #16 AND #12 1451

**Cochrane Library (2023-2-17)**

Search Name:

Date Run: 17/02/2023 23:02:45

Comment:

ID Search Hits

#1 (urticaria):ti,ab,kw (Word variations have been searched) 3194

#2 MeSH descriptor Urticaria explode all trees in MeSH products 52

#3 (hives):ti,ab,kw 308

#4 (#1 OR #2 OR #3) 3406

#5 "H1 antihistamine*":ti,ab,kw 202

#6 ("H1 antagonist*"):ti,ab,kw 163

#7 "H1 receptor antagonist*":ti,ab,kw 373

#8 MeSH descriptor Histamine H1 Antagonists explode all trees in MeSH products 8

#9 Bilastine or Ebastine or Fexofenadine in Title, Abstract or Keywords in all products 9051

#10 Loratadine or Desloratadine or Rupatadine in Title, Abstract or Keywords in all products 9633

#11 Levocetirizine or Cetirizine or Olopatadine in Title, Abstract or Keywords in all products 10018

#12 Mizolastine or Epinastine or Emedastine in Title, Abstract or Keywords in all products 8566

#13 bepotastine or Acrivastine or Azelastine in Title, Abstract or Keywords in all products 8881

#14 Astemizole or terfenadine or Clemastine or Setastine in Title, Abstract or Keywords in all products 9514

#15 MeSH descriptor Bilastine explode all trees 2

#16 MeSH descriptor Ebastine explode all trees 5

#17 MeSH descriptor Fexofenadine explode all trees 10

#18 MeSH descriptor Loratadine explode all trees 14

#19 MeSH descriptor Desloratadine explode all trees 5

#20 MeSH descriptor Rupatadine explode all trees 3

#21 MeSH descriptor Levocetirizine explode all trees 9

#22 MeSH descriptor Cetirizine explode all trees 15

#23 MeSH descriptor Olopatadine explode all trees 5

#24 MeSH descriptor Mizolastine explode all trees 7

#25 MeSH descriptor Epinastine explode all trees 1

#26 MeSH descriptor Emedastine explode all trees 2

#27 MeSH descriptor bepotastine explode all trees 0

#28 MeSH descriptor Acrivastine explode all trees 6

#29 MeSH descriptor Azelastine explode all trees 6

#30 MeSH descriptor Astemizole explode all trees 8

#31 MeSH descriptor terfenadine explode all trees 9

#32 MeSH descriptor Clemastine explode all trees 10

#33 MeSH descriptor Setastine explode all trees 0

#34 (#5 or #6 or #7 or #8 or #9 or #10 or #11 or #12 or #13 or #14 or #15 or #16 or #17 or #18 or #19 or #20 or #21 or #22 or #23 or #24 or #25 or #26 or #27 or #28 or #29 or #30 or #31 or #32 or #33) 13142

#35 (#4 and #34) 846

**PsycINFO (Ovid) <1806 to February Week 1 2023> （2023-2-17）**

1 double-blind.tw. 24719

2 random$ assigned.tw. 40888

3 control.tw. 498214

4 1 or 2 or 3 540929

5 urticaria.ti,ab. 217

6 hives.ti,ab. 105

7 5 or 6 315

8 4 and 7 38
